# “I Kind of Want to Want”: Women Who Are Undecided About Becoming Mothers

**DOI:** 10.3389/fpsyg.2022.848384

**Published:** 2022-04-07

**Authors:** Orna Donath, Nitza Berkovitch, Dorit Segal-Engelchin

**Affiliations:** ^1^Department of Sociology and Anthropology, Ben-Gurion University of the Negev, Beersheba, Israel; ^2^Department of Social Work, Ben-Gurion University of the Negev, Beersheba, Israel

**Keywords:** women, reproduction decision-making, indecisiveness, motherhood, Israel

## Abstract

This study focuses on women who define themselves as being undecided about becoming mothers. It addresses the question of how these women navigate their lives between two main conflicting cultural directives and perceptions: pronatalism and familism entwined in perception of linear time on one hand; and individualism and its counterpart, the notion of flexible liquid society, on the other. The research is based on group meetings designated for these women, which were facilitated by the first author. Ten women participated in the study—of whom, most were heterosexual, half were single, and half were partnered. Data were collected using (1) questionnaires completed during individual interviews that preceded the group encounter; (2) transcripts of the discussions held during the ten group sessions; and (3) questions regarding the status of the women’s doubts about motherhood asked 4 years after participating in the group. Our findings expand the existing typology of women’s reproductive decision-making, and demonstrate how categories that are commonly perceived as binary intersect when one challenges the rigid classifications of “active decisions” and “passive decisions”; “motherhood” and “non-motherhood,” and “want to be a mother” and “do not want to be a mother.” The findings also suggest that after becoming mothers, women can change their maternal status from “non-mother” to “mother,” yet still continue to view themselves as indecisive regarding motherhood. Based on our findings, we will argue that while indecisiveness about motherhood derives from individualized neoliberal rhetoric, it simultaneously undermines that same rhetoric and contradicts the injunction to “know, to decide, to strive.” It opposes the expectation in post-feminist discourse, that women will make choices about their bodies and exert them, while also opposing the pronatalist rhetoric, and the temporal linear discourse positing that women should “move forward” toward motherhood along with the ticking of the “biological clock.” Whereas some women sought to resolve their indecisiveness, other women found that the indecisiveness leaves all options open in a manner that expands their boundaries of autonomy in a society that seeks to limit it.

## Introduction

Over recent decades, in many countries worldwide, there have been declines in fertility and in the percentages of women who give birth and are interested in becoming mothers (Rowland, 2007; OECD, 2019). Yet, for many women, motherhood is still considered the default. Women commonly ponder questions, such as “When will I be a mother?,” “How many children do I want to have?,” and “Do I want a boy or a girl?,” while rarely considering or discussing the questions of “Do I want to be a mother?” and “If so, why?.” This is due to the broader ideology of a heteronormative and pronatalist society, where the pinnacle of femininity is still conflated with the birth and rearing of biological children (De Beauvoir, 1949/1970; Tietjens-Meyers, 2001), and in which cultural images link motherhood with “normality,” along with depictions of the “biological clock” that pressures women to make such decisions “on time” (Tietjens-Meyers, 2001; Amir, 2005; Lahad, 2012). This common assumption that all women want to become mothers hardly allows them to deliberate and be indecisive toward motherhood.

The declining fertility rates at a time when prevailing perspectives still praise motherhood has prompted growing research interest in the structural contexts and factors associated with the fertility decline (e.g., Donath, 2011; Kreyenfeld and Konietzka, 2017). Additionally, an expanding micro-level scholarship is focused on the intentions, reasons, and desires involved in women’s decisions to become—and, more recently, also to not become—mothers (e.g., Settle and Brumley, 2014).

The literature describes various typologies with regard to women who decide not to become mothers and those who express uncertainty about motherhood. For example, following the pioneer research of Veevers (1980), Houseknecht (1979), and Tietjens-Meyers (2001) highlight two distinct groups among women who are not interested in becoming mothers: the **early articulators**, and the **postponers**. The “early articulators” are those who knew from an early age that they would never want to be mothers. The “postponers” are those who have not yet decided, and they can be divided into two subgroups: those who always imagined that they would be mothers but who postponed doing so due to their life circumstances (e.g., lack of stable relationships, lack of stable income, and health-physical situation), and those who have always felt undecided about motherhood.

Gillespie (2003) and Settle and Brumley (2014) propose an additional typology based on their studies on women who are not interested in becoming mothers: “active deciders” vs. “passive deciders.” These researchers define active deciders as women who are determined not to pursue motherhood. Some of the active deciders made this decision at an early stage of their lives, while others believed they would eventually become mothers but changed their minds later. “Passive deciders” are also divided into two groups: women who are indecisive about motherhood, and women who desire to become mothers but do not pursue motherhood due to their life circumstances. The alternative term “fence sitters” was proposed by participants in Nandy’s (2017) study of women in India who were undecided about motherhood, while Martin (2021) referred to these women as “debaters.”

Women who are uncertain and are still deliberating about motherhood have not attracted much research attention. Two studies have addressed this unique group of women as a part of the larger category of ‘not having children’ alongside other subcategories: delaying motherhood, deciding against it, and not being able to do it (Kemkes-Grottenthaler, 2003; Martin, 2021). As such, they do not provide sufficient insight into the inner world of the “debaters.” Two additional studies have specifically focused on women who are undecided about motherhood, yet both were still limited in their scope. One is a personal account of Kelly Guyotte (2018), who explored her own ambivalence about motherhood. The other study (Barnett, 2016), conducted in England, investigated the experience of five women in their late thirties who are undecided about motherhood. This study shows that both participants’ age, which marked their decreasing fertility, and societal attitudes toward motherhood played a key role in shaping their experience. There is a need for research that focuses on the experience of younger women living in different sociocultural contexts who are indecisive about motherhood. Our study seeks to address this void. Also, little scholarly attention has been paid to the wider social, cultural, and ideological contexts, including contradictions and incongruences, within which women’s views, desires, and deliberation are embedded. We believe that these contexts are crucial for understanding them and, therefore, they stand at the heart of our study.

Women who are deliberating about motherhood have also received little attention in the public discourse. In the media, women who become mothers are frequently represented as the normative model that is taken for granted, and women who are not mothers and do not want to be mothers have been appearing with increasing frequency, although they are still a curiosity that is more appropriate for colorful write-ups. Meanwhile, the voices of women who are undecided about motherhood have hardly been represented in the popular and social media. One exception is a book published in 2016, *Motherhood: Is it for Me? Your Step-by-Step Guide to Clarity*. One of the authors of this book holds online group sessions for women who are indecisive about becoming mothers (Carlini and Davidman, 2016).

In the present article, we seek to expand the limited available knowledge by focusing on women who are undecided and introspective about their motherhood desires. This state of being undecided provides a unique lens through which to view some of the cultural principles that organize women’s lives and perspectives regarding family and reproduction—including linear time, social flexibility, and individualism on one hand, and pronatalism and familism on the other. Women’s uncertainty regarding motherhood is particularly interesting in the Israeli context, wherein alongside processes of individualization, familism and pronatalism maintain centrality at both the individual and collective levels (Lavee and Katz, 2003; Fogiel-Bijaoui, 2020). The current article addresses the question “How do women in Israel who are undecided about motherhood navigate their lives between these conflicting cultural perceptions as they maneuver among them, taking some into account and opposing others, all at the same time?” More concretely, the article presents a study focused on a group—the first of its kind in Israel—that was designated for women who define themselves as being indecisive toward motherhood. This group, facilitated by the first author, was formed to achieve three main goals: (1) to provide an introspective space for the women at both the individual and group levels, in which they would be able to examine the meaning of their uncertainty, as a means of assisting them in their reproductive decision-making process; (2) to create a new body of knowledge regarding women’s uncertainty about motherhood in the current sociocultural context; and (3) to make space for and provide legitimacy to indecisiveness about motherhood in Israeli society, where a women’s desire for motherhood is currently presented as unequivocal and comprehensive.

Based on the findings, we will argue that whereas the decision to forgo having children challenges the pronatalist hegemony (which lauds parenthood, especially motherhood, and reproduction), being in a state of indecision, ambivalence, and inactiveness with regard to reproduction challenges both the pronatalist social order and the neoliberal model (which emphasizes individuals’ freedom to choose and to act according to their wishes). We will also claim that indecisiveness toward motherhood, which involves taking one’s time, undermines the linear view of time that emphasizes the importance of moving forward and “developing” according to a culturally mandated biographical schedule (Lahad, 2016; Israeli-Nevo, 2017).

In other words, in this article, we propose a sociological discussion of the ways in which indecisiveness regarding motherhood relies on a neoliberal and an individualistic rhetoric, while simultaneously undermining such rhetoric and exposing the power of pronatalist and heteronormative social arrangements from an alternative perspective.

### Changing Family Institution and Reproductive Patterns

In contrast to the conceptualization of contemporary society as maintaining its rigid patriarchal and gendered structure, some contend that the social institutions, including the family, are fluid and constantly changing (Bauman, 2013). Traditional forms of the family have collapsed and been replaced by a multiplicity of forms (Beck and Beck-Gernsheim, 2002), including the possibility of opting out of any form of a family.

One major factor underlying these changes is the process of individualization that prioritizes the individual’s interest and needs in the name of “freedom of choice”—including the ability to choose a type of family that can guarantee self-actualization, intimacy, and freedom (Illouz, 2008), and the ability to leave relationships once they no longer satisfy one’s emotional needs and to pursue new relationships (Giddens, 1991; Beck and Beck-Gernsheim, 1995; Illouz, 2007, 2008; Garrett et al., 2016). Along with the individualization process, we can discern the emergence of the logics of capitalism and neoliberal free-market logics of action that have penetrated and dominated other areas of life, including the personal and family domains (Rose, 1996; Brown, 2003). These include the availability of contraceptive methods; increased women’s education; employment-related considerations; and challenges to previous social perspectives of “intimacy,” “familism,” and “femininity” (Maher and Saugeres, 2007; Bell, 2019).

Demographic statistics also attest to the changing face of the family. Alongside increased divorce rates, we also witness declining marriage rates, increased ages at first marriage and at the birth of one’s first child, and increased rates of paid employment among women from various social groups. These changes, combined with new reproductive technologies, have created new patterns of parenthood, as well as diverse family forms that are not contingent on the presence of a married or a cohabiting couple or of opposite-sex parental figures (e.g., Cliquet, 2003; Fincham and Beach, 2010).

Concomitant with the above-mentioned developments, we are also witnessing declining birth rates in many countries worldwide (Miettinen et al., 2015; OECD, 2019). Due to differences in broader institutional, cultural, and economic contexts, studies have also documented variations in fertility rates among different countries (Miettinen et al., 2015; Kreyenfeld and Konietzka, 2017). Given the rising number of young people who are not sure whether they want to become parents, it is assumed that the fertility rates will further decline (Sobotka and Testa, 2008). The recent use of the term “childfree,” which has replaced the term “childless,” reflects the current social perspective that one may make an intentional active decision to be a non-parent. Blackstone and Stewart (2012) relate to the distinction between the framework of “childless” and “childfree,” stressing that the former, which was prevalent in early research, had a negative connotation. Later work has created a shift to a “childless-by-choice” or “childfree” framework, emphasizing that for some, not being parents is an explicit and intentional decision rather than “an accident” and, therefore, the term “childfree” is a more accurate expression of the choice it describes.

Although “childless” and “childfree” are the common terms used to address subjects who do not want to be parents, in this article we intentionally use the framework of “women who do not want to be mothers,” when relating to participants who have considered the option of remaining non-mothers, as part of their deliberations. We used this framework for two main reasons: First, our aim is to stress that at the center of the debate resides the indecisiveness about motherhood and not about children in themselves. Second, while there are women who are emotionally uninterested in being mothers and who would prefer to avoid any relationship or quotidian interaction with children, there are also women who do not wish to be mothers but *are* interested in the company of children and, therefore, turn to therapeutic or educational professions in which they can work with children, or spend time with nephews, nieces, or other children within their families as well as with their friends’ children (Donath, 2013). These diverse relationships with children without giving birth to them nor raising them as their mothers, raise the question of whether “childfree” is the accurate terminology, as they do have children involved in their lives. This issue should be further elaborated in future studies.

### The Politics of Indecisiveness About Fertility and Motherhood

In light of, and as part of, the above-described changes, many women have continued to become mothers while being impacted by a closed ideological circle—in which the view of transition to motherhood moves from a biological-deterministic matter of fate that inevitably “programs” females to desire motherhood, to a matter of free will where women are the sole masters of their fate, and are internally and personally motivated to become mothers.

Feminist writers and activists have proposed a critical reading of the biological-determinist perspective (i.e., De Beauvoir, 1949/1970; Firestone, 1970; Gillespie, 1999; Donath, 2015a; Morison et al., 2015; Bell, 2019). However, the other interpretation—which reflects the spirit of the neoliberal capitalist era, and argues that a woman’s lifestyle is determined freely and that women are gaining increasing control over their lives and bodies—is not sufficient. This interpretation disregards the fact that the transition to motherhood is shaped by social, ideological, and hierarchical structures that limit many women’s spaces of autonomy (Himmelweit, 1988; Rothman, 1989/2000; Ginsburg and Rapp, 1991; Snitow, 1992; Tietjens-Meyers, 2001). These structures reproduce the equation of femininity with motherhood, thus ingraining the view that establishing a family through childbirth/adoption is the only and most meaningful rite of passage for societal recognition, and an almost exclusive metric for the quality of a woman and her life (Tietjens-Meyers, 2001).

Women’s limited reproductive autonomy—including their limited autonomy to identify their presence of absence of desire for motherhood—has also been manifested in social responses that denounce non-motherhood. Although these responses have been diminishing over recent years, they still exist and still tarnish the personalities and lives of women who do not want to be mothers. Many women who are not interested in becoming mothers are still viewed as “unreal women”; as selfish and childish; as “strange birds”; as damaged, hedonist, and pathetic; as betraying their families; as objects to be pitied; and as failing to achieve the most valuable accomplishment of women in this world (Rich, 1976; Gillespie, 1999; Donath, 2011; Morison et al., 2015). Within this context, even if more and more women from various social groups have greater autonomy over reproductive decision-making, many may continue to have vague attitudes toward motherhood, lacking the possibility to explore their own desires and capabilities and to accordingly reach a decision regarding motherhood.

The social opposition to uncertainty regarding motherhood, and women’s limited ability to identify their motherhood-related desires, are the outcomes of an ideological mix that combines pronatalism and heteronormativity with other social logics related to neoliberalism and individualism, social perceptions of time, and subjectivity, among other components.

### Neoliberalism and Individualism

The concept of the individual self is central to the neoliberal perspective, in which the individual is the ideal proponent of autonomy. In this perspective, individuals are considered to have free will with regard to their chosen paths of life; are perceived as rational and calculating and exclusively responsible for the outcomes of their choices; and are expected to fulfill their needs and desires (Bauman, 2001; Beck and Beck-Gernsheim, 2002).

While the life course was previously based on anticipated transitions from one stage to another, this has been replaced with what Beck (1992) refers to as “choice biography,” which should allow a person to “be themself” and “realize their potential,” as indicated by Rose (1990, 1996). Without clear and obligatory rules for the arrangement of life stages and the transitions between them, there are more and more junctures at which people must make decisions and construct their biographies, which Woodman (2009) referred to as the “do-it-yourself biography.” Ideally, people who can optimally function within a biography arranged in this manner are the neoliberal subjects—the “subjects who choose,” the “subjects who know their goals, who are aware of their preferences, who are able to make rational decisions, and who act in accordance with their decisions” (Gill, 2008; Gill and Scharff, 2011; Rottenberg, 2018).

Within the integration of pronatalism with neoliberalism and individualism ideologies—in the name of neoliberal rhetoric, where “humans are masters of their fate and write their own biographies”—there are many social arenas that foster skills for creating autonomy, that help people practice those skills, and that grant them the legitimacy to use those skills, through breaking down and reconstructing social perspectives, and through personal and collective social perspectives. However, this autonomy is relatively limited in the realms of reproduction and childbirth. Despite the growing number of women who have decided not to pursue motherhood, they remain a relatively small group. OECD data indicate that the percentage of these women ranges from 10 to 20% and is even less than 10% in quite a few countries (OECD, 2019). This may suggest that most women do not engage in an actual decision-making process on the subject of motherhood.

### Time and Subjectivity

The fertility and birth arena has usually been addressed as part of the discourse on the rhythm of a “ticking clock.” Young women learn that there is a “biological clock” that limits their time span for becoming mothers and that they are expected to unhesitatingly organize their lives according to the schedules of this clock.

As shown earlier, along with these “natural” timelines, women are required to follow additional timelines in a social reality dominated by the discourses of individualism, neoliberalism, and capitalism, and in which the ethos of “conquest,” “attaining goals,” and “decision-making” prevail in the immediate term. All of these clocks lead to the standardization and homogenization of a road map of the “natural and proper way of life,” which includes specific stations that people must pass through over time: from educational attainment, to employment, to marriage, and to parenthood (Amir, 2005; Elchardus and Smits, 2006; Lahad, 2009).

According to Davies (1990), this kind of time perception divides human experience into discrete and consecutive units, where a person “progresses” from one unit to another. This logic also determines the “proper” life course, and what one must do to follow it, at the right time and the right pace, in order to develop in the “right” linear direction (Amir, 2005; Lahad, 2009). Roth (1963) argued that most people ensure their movement at the correct pace and in the correct direction based on a normative timeline established by a group, which serves as a measure of their progress. Through comparison with that norm, people can interpret their progress and determine whether they are lagging or progressing appropriately, as well as how they should feel in order to keep up the pace and fall into line.

Within this social reality, women’s indecisiveness about motherhood can be interpreted as a blatant violation of timelines—both “natural” and social—and as an unwarranted delay. Irrespective of issues related to reproduction and motherhood, research indicates that waiting tends to be associated with characteristics of hopelessness, lack of strength, lack of productivity, being stuck, distress, and vulnerability. Waiting is also viewed as a sign of inactivity and as passively being at the mercy of time, which will “already do what it wants with us,” or as resulting from an expectation that “something is about to happen” (Lahad, 2016; Israeli-Nevo, 2017). These characteristics contradict the ethos of individualism, neoliberalism, and capitalism, according to which humans are active agents who are supposed to write their own biographies and take their fate into their own hands, to navigate their lives with confidence and tenacity toward the “right goal” at the “right pace.”

Clearly, there are contradictions and tension between the logics of neoliberalism and individualism and the logics of linear time. The first logic conveys the message that “everything is fluid and open” for the neoliberal subject. In contrast, the second logic sets limitations, stations, and timelines, which restrict the subject’s freedom of movement and the amount of time spent at each “station” when writing one’s own biography.

### The Israeli Context: Autonomy Is Especially Limited

As mentioned earlier, Israeli society provides a particularly interesting context in which to examine reproductive decisions. This is because along with the processes of individualization and the significant changes witnessed with respect to family patterns during the past few decades, Israeli society has remained a familistic and pronatalist society that lauds motherhood (Fogiel-Bijaoui and Rutlinger-Reiner, 2013; Berkovitch and Manor, 2022).

The high level of familism in Israeli society is reflected in its higher marriage rates, lower rates of divorce and non-marital births, and lower age at marriage compared to most OECD countries (Central Bureau of Statistics (CBS), 2020). Israel has the highest fertility rate among all 35 OECD countries, as well as among a large number of developing countries and many Middle Eastern countries (Weinreb et al., 2018). This high fertility rate is related to cultural forces that praise children, as revealed in a study showing that the vast majority of the Israeli public considers children to be a source of happiness and blessings, and a major contributor to meaning in life. Notably, over 50% of the respondents believed that the lives of people who have never had children are empty and meaningless (Glickman et al., 2003).

The high fertility rate in Israel is also related to the country’s status as a superpower of assisted reproduction, which imposes very few limitations on the availability and subsidized use of these technologies (Shalev and Gooldin, 2006; Birenbaum-Carmeli, 2016). The availability of assisted reproduction technologies enables many women—including those who have postponed their first birth to later ages—to become mothers and fulfill the commandment to “be fruitful and multiply.” The Ideological validity of this Biblical commandment among both Orthodox and secular Jews is usually attributed to the traumas of the Holocaust as well as the Israeli–Palestinian conflict (Herzog, 2004; Birenbaum-Carmeli, 2016), accompanied by fear of undermining the demographic balance vis-à-vis the Palestinians. Within this context, reproduction has become a tool in the national struggle (Berkovitch, 1997; Shalev and Gooldin, 2006). As such, it has been mobilized as justification of women’s right to demand state funding of fertility treatments to achieve motherhood (Gooldin, 2008).

Nonetheless, as mentioned, Israeli society has been undergoing processes of individualization and neoliberalization (Filc and Ram, 2004; Fogiel-Bijaoui, 2005; Goodman and Tavori, 2010; Ram, 2013). These processes are reflected in the family institution among most social groups. Family roles have become blurred and are more subject to interpretation than they were in the past, and people are less bound to traditional arrangements and collective considerations as they choose the path and pace of family life that are appropriate for them (Fogiel-Bijaoui, 2020).^1^

In contrast to in other societies, the processes of individualization in Israeli society do not necessarily contradict family and familism but have rather become another means of strengthening these aspects (Berkovitch and Manor, 2022). For example, the intensive struggles of gay men and lesbians to become part of mainstream society have focused on recognizing their rights to couplehood and a family, particularly their right to parenthood (Kama, 2011). Family and birth have maintained their status not only for collective reasons but also for individualistic reasons related to the neoliberal discourse on rights or, more specifically, on the “emotional right to happiness.” In that context, Sigal Gooldin (2008) argued that family life in general and parenthood are currently perceived as the key to happiness and self-fulfillment.

The processes of neoliberalism in Israeli society, similar to the processes of individualism, do not weaken the institution of the family nor do they challenge the centrality of motherhood. This is reflected in research investigating how low-income mothers in Israel maneuver between seemingly contradictory dictates of neoliberalism and motherhood between care work and paid work. The findings reveal the ways by which these mothers mobilize elements of the neoliberal discourse and at the same time challenge it by entwining it with the maternal discourse to create their own model of the good mother (e.g., Lavee, 2016; Meler, 2016; Sa’ar, 2016; Herbst-Debby, 2018).

### The Current Study

In the current study, we sought to explore the experience of indecisiveness toward motherhood within the context of the above-described contemporary cultural perceptions. We, therefore, used a phenomenological approach, which focuses on the meanings ascribed by a particular group of people to the experience under study (Grossoehme, 2014). The specific aims of the study were to enhance our understanding of the issues and dilemmas involved in Israeli women’s uncertainty about motherhood and to examine the meanings and experiences of participation in the presently studied pioneer group.

## Materials and Methods

### Procedure

After obtaining ethical approval from the departmental ethics committee at Ben-Gurion University of the Negev, a call for participation was published, announcing the opening of a group designated for women who are indecisive about motherhood, with meetings planned to be held at the university. Within this call for participation, it was emphasized that the group was not intended to provide “right” answers to the issue of motherhood, but rather to enable each participant to identify the option most suitable for her. It was also noted that the group was a part of a research project that would be based on material derived from the group sessions. In addition, it was specified that identifying details of the group participants would remain confidential and that the women would be able to discontinue their participation in the group at any stage they chose.

Following publication of the call for participation, 20 women inquired about the group. After receiving additional information, 11 women expressed willingness to participate in the group. As part of the pre-group preparation process, during the weeks preceding the first group session, individual interviews were scheduled with the 11 women who expressed willingness to join the group. During these interviews, all women signed consent forms. One woman discontinued her participation after the first group session. All of the group sessions were videotaped, with the participants’ permission, and fully transcribed.

### Data Collection and Analysis

The 10 group sessions attended by the participants served as the main source of data collection. This method is similar to a group interview. While individual interviews adopt an overly individualistic approach, which isolates individuals and examines attitudes and behavior outside of a social context, group interviews create a shared social space for people to communicate with each other (Montell, 1999). This space allows for observation of dynamic negotiations and of the process of constructing shared meanings and narratives deriving from participants’ reactions to each other (Lavie-Ajayi, 2014; Kook et al., 2019).

Data collection was also based on three additional components. First, a questionnaire, consisting of demographic questions and three open questions, was completed by the participants during the individual interviews conducted prior to the group sessions. After completing the demographic questions, the women were asked to write down their answers to three open-ended questions that were intended to help plan the group sessions: (1) How is a group dealing with indecisiveness about motherhood relevant to you?; (2) What are your expectations from participation in this group?; and (3) What will make your participation in the group meaningful for you? Second, at the end of the last group session, the women were asked to complete a feedback questionnaire designed to explore the meaning of participation in the group for the participants and to learn whether and how group attendance had helped them understand and/or feel their indecisiveness differently than they did in the past. Third, to examine the status of participants’ indecisiveness from a time perspective, at 4 years after group termination, the group facilitator contacted all participants by e-mail and asked them to indicate their current family status and self-defined status regarding their indecisiveness toward motherhood. The participants were aware that their e-mails would form part of the data collection and gave their consent to it.

The data reported in this article are based on three components (e.g., written answers to the three open-ended questions, transcripts of group discussions, and e-mail correspondences) and were analyzed using thematic analysis (Braun and Clarke, 2006). In the first stage, the authors read and re-read all the group interview transcripts and the participants’ written responses to the questions noted above in order to become familiar with the various aspects of the data and to identify initial ideas for coding. In the second stage, initial codes were created and matched with data extracts. In the third stage, the initial codes were sorted into potential themes and relevant coded data extracts were collated within each potential theme. In the fourth stage, all the identified themes were reviewed and were examined in relation to both the coded extracts and the whole data set. Finally, all themes were defined and named.

In the current article, we will present the three major themes that emerged from our analysis: (1) Motivations for participating in the group; (2) Typologies of indecisiveness; and (3) Advantages and disadvantages of remaining in a state of indecisiveness about motherhood.

### Participants

The participants were ten women who attended at least nine of the ten group sessions. These women were recruited through a call for participation that was distributed *via* the authors’ university website, online forums for women, the first authors’ blog, and through therapists working in the southern region where the group was held.

The participants ranged from 25 to 41 years of age. All were Israeli-born Jewish women. Three were of Middle Eastern or North African origin (“Mizrahi”), six were of European or American origin, and one was of mixed origin (“Ashkenazi”). At the time the group sessions were held, five of the women were single, three were cohabitating, and two were married. Among the ten participants, two lived in a kind of commune as part of a shared group dealing with education. Five participants defined themselves as secular, one as atheist, one as religious, and three chose not to define themselves in that context. Eight of the women defined their sexual identity as heterosexual and one as asexual, and one mentioned that she does not have a definitive identity.^2^

Before writing the article, all participants were invited to choose the name under which their quotes would appear. Several of them asked to be quoted under their real name, others chose a pseudonym.

### Description of the Group Sessions

In total, ten group sessions were held: one group session once a week, for 10 weeks, each lasting about 90 min. The first session was devoted to helping participants get to know each other, and to creating a group contract based on participants’ expectations of the group. Sessions 2–9 begun with an “open space” within which the participants were invited to bring up issues that concerned them and/or to share the experiences they encountered since the last group session. After this part of the session—which lasted 10–20 min, the rest of the session was devoted to discussions among the participants about topics selected by the group facilitator, based on her research findings regarding reproduction and (non)motherhood (Donath, 2011, 2015b) as well as on participants’ answers to the open-ended questions they addressed during the individual interviews. These topics dealt with issues inherent to indecisiveness about motherhood—such as fear of how one’s surroundings will respond to one’s decision to be a non-mother and possible ways of coping with that fear; regrets about remaining a non-mother and regrets about the transition to motherhood; and the distinction between “wanting” and “consenting” regarding reproduction and motherhood. Although these sections were structured, there was room for the participants to take the issues discussed to different directions in the discussions. The last group session was devoted to summarizing the previous sessions, as well as to various aspects related to parting from the group—both from the perspective of indecisiveness and from the perspective of the sessions themselves.

### Ethical Consideration

In light of the pronatalist ideology characterizing Israeli society, the group establishment was accompanied by an ethical issue—namely, what does it mean that the group facilitator was a woman who does not want to be a mother, and who publicly identifies with that position? It can be reasonably assumed that if the group facilitator had been a mother, she would not have been “suspected” of spreading pro-birth propaganda among the group members. In contrast, a facilitator who is not a mother herself can easily be suspected of disseminating propaganda. In light of this, the facilitator confronted the group members with this issue during the individual interviews, as well as during the first group session, in which participants’ expectations of the group were discussed and it was emphasized that the group sessions would not serve as a forum to preach against motherhood. It was further clarified to the participants that the aim of the group was not to create uniformity among the women, but rather to acknowledge the diversity within the group. Nonetheless, the sessions were held with the facilitator’s constant awareness of her personal and public positions and their potential to influence the participants.

## Findings and Discussion

### Motivations for Participating in the Group: “I Do Not Know Anyone Like Me Except Myself”

As mentioned earlier, during the individual interviews, participants were asked to complete a brief questionnaire in which one of the questions was: “How is a group dealing with indecisiveness about motherhood relevant to you?” The following are some of the offered responses:

**Dalit (age 31, single):** *I put the question of motherhood aside for a long time. It wasn’t clear to me whether I wanted to be a mother. I reopened the question one or two years ago, and I’m still uncertain about it today. I’ve been trying to distinguish what I want to do from the things that I’m influenced by in society, but they really can't be distinguished*.

**Abigail (age 34, married):** *I’ve been indecisive for years […] Outside of the web, there’s no place for uncertainty… It’s either virtual, or at home with your partner*.

**Noga (age 32, married):** *I’m interested in talking to or hearing other women who might share my views on motherhood. At the moment, I do not know anyone like me except myself*.

**Inbal (age 27, partnered):** *I’m currently debating about that very question [question regarding motherhood], and most of the women that I know hold a specific opinion about this issue. I’m interested in participating in a framework that objectively examines the issue*.

The importance of a group dealing with indecisiveness about motherhood also emerged in the group sessions. For example, during the first group session—in which participants were invited to share their expectations of themselves, the group process, the group members, and the facilitator—Inbal and Rotem said the following:

**Inbal (age 27, partnered):** *On the whole, my expectation is that contrary to the opinions I’ve been hearing from my friends—who hold very specific opinions that I am familiar with—I will be hearing here many different opinions and that I will understand where you come from…. Maybe something that one of you will say will provide me with a tool and direct me in a direction that is more appropriate for me. Just to hear as many of you as possible, and that you will be as open and sincere as possible…. I believe this will help me and all of us, and that it will lead to positive outcomes in general…*

**Rotem (age 30, single):** *I feel that most of the pressure—at least the pressure that **I** face—regarding pregnancy is from the “women’s tribe.” Especially among Mizrahim [those whose origin is in the Middle East and North Africa] and among those in the middle class to which **I** belong, and it’s very **good** for me to see the wide variety here*.^3^

The above quotes demonstrate how the hegemonic discourse, which tends to isolate women from one another, led many participants to believe that they were the only ones who were uncertain regarding their will to become mothers. Consequently, they may have been strongly influenced by their partners or by society, while downplaying their own desires and needs. It appears that participants’ willingness to participate in the group derived partly from the realization that there were other women debating motherhood, and the hope that by meeting these women and hearing their perspectives, they might better understand the questions that they were asking themselves.

Based on the participants’ statements regarding their motivations for participating in the group sessions, we can understand that the heteronormative and pronatalist discourse—similar to other hegemonic discourses—operates by “naturalizing” the “standard” life course, as well as the transition from one stage to the next. In the current context, the transition to motherhood is perceived as a natural step that does not require thought or discretion because women are naturally endowed with a “maternal instinct,” and with “feminine traits” that are associated with caring for others. Thus, even if many of the participants did not express opposition to motherhood, they did oppose the notion that motherhood is a command that does not have to be decided on and that does not require thought, deliberation, or discussion among women themselves.

Naomi (age 27, partnered) made the following statement: *“I define myself as a woman who is not interested in having children. Over time, my partner has begun to think differently than me, and I’m afraid that I will eventually be persuaded by him without giving the matter serious thought*.*”* This also reflected the concerns of other participants—that regardless of what they ultimately decided, they mainly sought to ensure that they were not naturally swept into motherhood. Rather, they wanted to make sure that they—as we term it—“turned every stone over” before making a decision. The meaning of “turning over every stone” differs according to the lens through which it is viewed. Through a neoliberal lens, participants’ determination to “turn over every stone” may be perceived as a reflection of the neoliberal subject who is capable of fulfilling her autonomous choices and of taking full responsibility for her wellbeing (Rottenberg, 2018). However, through a pronatalist lens, the women’s intention to “turn over every stone” in their reproductive decision-making may be perceived as challenging the societal expectations that they will “automatically” become mothers, given that as “females,” they are inherently coded with a desire to give birth and raise children. This does not leave women an option to view the transition to motherhood as a decision (Donath, 2015a,b). By making the “right” decision, they will be simultaneously following these two conflicting cultural stances, as they will be preserving the pronatalist directive in the neoliberal era. Nevertheless, as we will demonstrate later, the participants’ state of indecisiveness about motherhood undermines both of these cultural directives—as they are expected to reach a decision and to do so immediately.

### Typologies of Indecisiveness: “I Do Not Want to Be a Mother, But I’m Still Debating Whether or Not to Become a Mother”

Our findings suggest that the participants did not reject and/or were not undecided about motherhood as a social institution. Rather, they were in a state of indecisiveness about their own mothering. Although they sometimes oscillated between the two modes, they mainly referred to their own reproductive deliberations and wishes.

During the sixth session, it became clear that women face complex possibilities. At this point, the group members were well aware of the meaning that each participant attributed to her unique experience of indecisiveness. From the group discussion conducted in this session, emerged a typology that differs from those proposed in the literature:

(1) **I know that I want to be a mother, but am still debating/considering whether or not to do so** (e.g., due to fears about the implications of motherhood and due to life circumstances that the woman does not perceive as optimal for raising children)
(2) **I know that I do not want to be a mother, but am still debating/considering whether or not to become a mother** (e.g., due to pressure from the woman’s surroundings, the stigma of non-motherhood, fear of feeling alienated, and uncertainty regarding old age)
(3) **I do not know if I want to be a mother or remain a non-mother.**

The participants formulated these possibilities following a statement by Noga (age 32, married): *“I feel like **I do not want [to be a mother]**. Completely. And yet I’m still undecided.”* This statement became even clearer in the ninth group session, when the following discussion took place:

**Noga (age 32, married):** *I want to ask you something because I’m so confused now. I recently understood that I am no longer uncertain. I understood that I **do not want [to be a mother].** But then I understood that this is not the issue that I am debating. I am debating two: whether or not I want to be a mother—and I’ve decided that I do not want to. But I’m still debating whether or not I’m going to do it [become a mother] [there are voices in the room saying “Wow,” and heads are nodding]. I may go through with it even though I’ve decided that I do not want to. So now I do not know how to answer all those questions*.

**Rotem (age 30, single):** *You just cracked us up!*

**Abigail (age 34, married): *You are saying*** *the same thing Nitzan said*.

**Nitzan (age 33, partnered):** *Yes. Totally*.

**Group Facilitator:** *It appears that indecisiveness includes quite a few layers*.

*One word that is based on many layers. Nitzan, I noticed that Noga’s words helped you organize your thoughts*.

**Nitzan (age 33, partnered):** *It sent a “ting” through me [she makes a hand motion indicating that she finally understood what was going on]*.

**Inbal (age 27, partnered):** *I really identified with what Noga said, that I do not want [to be a mother], but it’s still possible that I will go through with it. It was interesting to listen*.

A week later, in the last group session, the following statements were made:

**Nitzan (age 33, partnered):** *I went **all** last week with a giant **light** on my head. Ting! I was asked three times during this week, “Well, what about children?” And then I mentioned the conclusion I had reached and felt very good about myself. Generally, what I said was that I **know** I do not want [to be a mother] but now I’m debating whether to do it anyway*.

**Dalit (age 31, single):** *That’s what you said when people asked you?*

**Nitzan:** *I really, really feel that it’s … it’s just a great answer*.

**Facilitator:** *How did they react?*

**Nitzan:** *Of course, the first thing I have to do is apologize, and explain, and expand … and they should not pity you for doing something you do not want to do and all that, but … I say that it really releases me. I think that this answer gives me a lot of freedom*.

**Abigail (age 34, married):** *It would be really interesting to know whether the people around you think it’s **preferable** that you will do something you **do not** want to do [become a mother]. To what extent is the condition that one must have children a strong one?*

**Nitzan (age 33, partnered):** *Ah, it depends on who you ask*.

**Abigail:** *Has anyone said to you: “Great!” [she claps her hands] “Way to go!?”*

**Nitzan**: *No one will say “Great!” It’s mainly “Oy.” It’s a matter of “Oy, why do not you want children?,” but on the other hand, it’s “Oy that you are going to maybe do something you do not want to do.” It’s “Oy” in any case, as far as people are concerned*.

These above quotes demonstrate how participants had negotiated the meanings of their indecisiveness and constructed their individual narrative of indecisiveness during group discussions. The quotes call for deeper examination of the situation in which women may become mothers against their will. Previous studies (Donath, 2015a) have shown that women who do not want to become mothers can sometimes be exposed to attempts at persuasion, in and outside of the home, that make it difficult for them to be consistent about living as they wish and according to what they know about themselves. Some of these women must cope with a reality in which, almost every day, they have to stave off direct or indirect attempts at persuasion to become mothers against their will. Thus, even if a subject is said to have written her own biography that includes the decision to become a mother, the entire process raises questions about women’s autonomy, as part of a couple or as a member of society.

In contrast to this situation, Nitzan, who became a mother several years after the group sessions ended, described a different event. Although her partner’s attitude differed from hers because he wanted to be a father, he did not attempt to persuade her, but enabled her to make a decision about whether or not she would become a mother even though she did not want to be. Nitzan’s decision may be confusing and may arouse pity if one interprets it to mean that she was forced to decide. However, during the group sessions and to this very day, she has insisted that she made the most complex use of the essence of autonomy. She believes that it is completely possible to combine not wanting to be a mother with the joy related to both her spousal relationship and the upbringing of her daughter. This joy was partly made possible by the feeling that she owns knowledge of herself, and by the understanding that her continuous lack of desire to be a mother is respected, even when her status has changed to “mother.” This is what allows Nitzan to contain and recognize all these contradictions, without deleting any option or ignoring any feeling.

The possibility that women will become mothers and still define themselves as undecided about motherhood also emerged in the e-mail correspondences conducted between the group facilitator and the participants, 4 years after the group termination. At this time, the participants were asked to indicate their current family status and their self-defined status regarding their indecisiveness toward motherhood. Due to loss of contact with one of the participants, only nine participants responded. Of these, six reported that they had not become mothers, and three reported that they had become mothers. Among the six participants who did not pursue motherhood, three noted that they are still debating motherhood, while the other three stated that they had decided to forgo motherhood. Of the three participants who had become mothers, two continued to define themselves as undecided:

**Nitzan (age 33, partnered):** *“This may surprise you. My answer to the question is that I’m no longer uncertain—but I maintain the identity of undecided (hope this sounds logical)*.*”*

**Noga (32, married):** *“At the time, I thought I might choose to do so [become a mother] even though I did not want to. In the end, I did not choose at all—it happened by chance … so it’s true that it may seem that there is no more uncertainty about whether to become a mother—it already happened. But because I never actually made a decision, and because to this day I cannot say what I would have chosen, I still define myself as undecided*.*”*

These statements indicate that the differentiation between “mothers” and “non-mothers” and between “I want to be a mother” and “I do not want to be a mother” is not binary. It can be flexible and can have many meanings in ways that do not necessarily correspond with the women’s self-definitions and attitudes toward motherhood, enabling women to cross-check and integrate categories that are perceived as rigidly binary. Thus, women can change their concrete status from “non-mothers” to “mothers” as they become mothers, and still define themselves as undecided with regard to motherhood.

Our findings also suggest that the women who participated in the current study cannot be divided into hermetic categories, such as “active deciders” and “passive deciders” (Gillespie, 1999; Settle and Brumley, 2014), because the very fact of their participation in the group sessions indicates that they all sought to clarify to themselves that they would not be making any decisions without “turning over every stone” in their decision-making process. We are not arguing that the wish to clarify and map their attitudes should be regarded as active decision-making. Rather, we suggest that the discourse regarding reproduction decision-making should refrain from the value judgments inherent in the labels “active/passive,” mainly due to the complexity of the intrapsychic processes involved in this decision-making.

### Advantages and Disadvantages of Remaining in a State of Indecisiveness About Motherhood: “I Kind of Want to Want”

The inability to decide whether to become a mother or remain a non-mother can have major impacts on a woman’s wellbeing and self-esteem, as well as on her status within her family, community, and society as a whole (Tietjens-Meyers, 2001). Thus, one might assume that many women who are deliberating motherhood seek to renounce their undecided status as soon as possible. However, our findings indicate that this assumption does not universally apply to all women. During the group sessions, several participants indeed expressed their desires to be released from the state of uncertainty, as well as to feel normal and belonging. Such desires were clearly expressed by Nitzan **(age 33, partnered)** during the first group session, when presenting her expectations of the group process:

*My expectation of this process … I have some sort of an internal expectation that by the end of the group process, I will **want** children, because that is much easier in our society. I kind of want to want [she smiles]*.

Nitzan’s statement is consistent with Maher and Saugeres’ (2007) findings that their participants wished to experience a desire or a strong urge for motherhood, which would assist them in their fertility decision-making. The finding that women who are not interested in becoming mothers may wish to develop a desire for motherhood indicates that—contrary to the message conveyed to women from an early age—not all women have a natural biological which is usually referred to as “maternal instinct.” Moreover, women’s wish to desire motherhood emphasizes the need to re-examine society’s rigid imperatives, which lead women to hope that they will eventually want to pursue something that they are not interested in pursuing. The finding that society’s rigid expectations play a critical role in shaping women’s reproductive decisions suggests that children are sometimes brought into the world merely because their mothers “succeeded” in forcing themselves to want to have children.

Along with the wish to be released from their state of indecisiveness, toward the end of the group sessions, the women also expressed reluctance to depart from their state of uncertainty. The following group discussion reveals the complex and dynamic negotiation between the group members with regard to the implications of their indecisiveness:

**Group facilitator:** *Does anyone want to say something about the advantages—if there are any—of uncertainty [regarding motherhood]? The advantages of defining yourself as being undecided*?

**Noga (age 32, married):** *The advantages of not deciding?*

**Facilitator:** *Yes*.

**Noga:** *Clearly. For all of us, it’s the **best** thing*.

**Facilitator:** *You’ll speak for yourself?*

**Noga**: *No. I am speaking on behalf of all women who call themselves “undecided” [about motherhood]. Being uncertain has the most advantages, because for women who define themselves as “undecided,” the implications of “yes” are difficult, as are the implications of “no”—so [indecisiveness is] the best place to be in that situation. The other two situations are too difficult*.

**Naomi (age 27, partnered)**: *As Nitzan said, you just do not take any responsibility*.

**Abigail (age 34, married):** *But that’s also not a pleasant place to be. I **hate** that place*.

**Daphna (age 41, single):** *Like Nitzan said, in the first session that she wants to want. I think that at least for me, if I decide not to [become a mother], I will be “marked” in some way. People will look at me. I’ll no longer be the one **who is uncertain** about having children. I’ll be the one who **decided** that she does not want children. What does that say about her? It’s preferable to want [to be a mother]. It’s [not wanting to pursue motherhood] a loaded issue*.

**Nitzan (age 33, partnered):** *When you are undecided, you are still on the margins of society. And if you decide not to [become a mother], then you become an outsider*.

**Facilitator:** *I can understand the difficulty of giving up the state of indecisiveness if there are implications for the understanding that I do not want to be a mother*.

**Abigail:** *The implications of bringing children into the world are much more scary [laughs]*.

**Facilitator:** *I do not know. I assume each of you has her own scale*.

**Abigail:** *I assume that everyone here believes she is strange and absurd—but we are already used to coping with that*.

**Facilitator:** *But maybe it’s like Daphna said, right now people are thinking that “you are [potentially] strange,” and there are three dots after that. It may be different than “you are strange” followed by an exclamation mark. I do not know. Maybe. Does anyone want to add anything about the advantages of remaining in a state of indecisiveness?*

**Abigail:** *It’s pleasant. It’s having your cake and eating it too. Not deciding enables me to have the option of sometimes thinking this way, and sometimes thinking that way. Whatever’s comfortable for me*.

**Facilitator:** *Do you wish to depart from this position?*

**Abigail:** *Sometimes. It’s very comfortable and pleasant. I have not reached a point yet where I can say, “I want to be a mother” or “I do not want to be a mother.” Sometimes it’s more in the direction of “I do not want [to be a mother]. But it’s comfortable for me to put an asterisk next to it of “at the moment*.*”*

**Nitzan:** *I think I said this at the last meeting, that there is something about the position of uncertainty that is a position in itself. This position does not just consist of not **deciding**, not **committing** oneself to any position—rather it’s a privileged position of talking about the issues from another viewpoint. I observe reality from a place that is neither here nor there, but somewhere else. Like it’s a little bit from on high. I feel that my self-perception as undecided is a full position; it enables me to look at issues—and at everything else—from a patronizing stance [people laugh]. There is something in the position **itself** that enables you to remain indecisive all your life. It’s really awesome. If it were not for the f-cking biological clock, it would be wonderful. And I understood that part of being indecisive may also be creating a reality in which I make **better** decisions—that maybe there’s more time, and in which it’s also possible to negotiate about the right time. And then if you continue with uncertainty about little things, like how many children you want* etc.*, it eases the major indecisiveness a bit […] I think I’m somewhat in the process of reclaiming the uncertainty—that the decision is **not** a whole set of things but it breaks down their meaning, and breaking down their meaning might mean taking something from here and something from there, perhaps trying something I devised myself [she makes a hand motion of mixing]*.

The above group discussion indicates that staying in a position of indecisiveness has quite a few advantages, in that society waits for them to demonstrate their commitment and act in accordance with the capitalist, neoliberal ethos of pursuing and reaching goals, as well as with the ideal type of the neoliberal individual, that is, “an individual who chooses.”

One advantage of remaining in a state of uncertainty that was mentioned by participants was that it enabled them to “stay under the radar” of intense social pressure and judgmentalism. In a society that tends to deride women who are not interested in becoming mothers,^4^ saying “I do not know yet” enables one to remain in a situation where one can stave off direct criticism. A statement of uncertainty could also reduce the likelihood that women who are debating motherhood are turned from being subjects **who ask themselves questions** to being the objects of questioning and subject to pleading and attempts at persuasion **by others.**

Some participant described another advantage of remaining in a state of uncertainty—that it enabled them to keep all options open, thereby expanding their boundaries of autonomy. Indecisiveness enables the imagination to run in all directions without needing to ground it, and without having to take action deriving from each decision. For example, if a woman decides to become a mother, she may need to stop using birth controls (if she will be pursuing motherhood within a heterosexual relationship), or to start investigating the option of sperm donation (if she will be pursuing motherhood in a non-heterosexual relationship or without a partner), or to go for gynecological examinations, start taking nutritional supplements to increase the chances of pregnancy, take economic considerations into account, etc. On the other hand, if a woman decides to remain a non-mother, she may need to make clear statements about her reproductive decision, which will require her to deal with the responses of her family, friends, and workplace. A woman’s decision to forgo motherhood may also change her view of romantic and sexual encounters and require her to reveal her reproductive intentions from the very outset. Additionally, she may need to reconsider her place of residence (e.g., a city, small town, or a neighborhood populated mainly by families with children), as well as reconsider staying in Israel, where being a non-mother is more openly criticized than in other countries, etc.

Analysis of the group discussions reveals that by leaving all options open, participants felt that they could break down their uncertainty into smaller units, which allowed them to address an array of questions, rather than just the question of whether they want to become a mother. In this respect, the state of indecisiveness paves the way for a detailed exploration of diverse questions related to motherhood, such as “Do I want to be a mother?,” “Whether the answer is yes or no, why?,” “Do I want to raise a child without a partner?,” “Am I prepared to be a mother, irrespective of the ‘ticking of the biological clock?’,” “Am I willing to be a mother because my partner is interested in having children even though I am not?,” “Do I want to arrange with my partner that he/she will bear the main responsibility for raising the child?,” “Can I imagine being able to not play an active role in raising the child if I did not want to be a mother from the outset?,” “What conditions will I need in order to become involved in raising the child?,” etc.

The complex roadmap that emerged from the group discussions also reveals that by leaving their options open, participants felt that they could control time. According to Amal Jamal (2008), “Dividing time, sorting it, and turning it into a reference tool reflect the power relations between groups of people. The subject who sorts imposes, through the division of his/her time, a certain type of relationship with others” (p. 354). Within the context of reproduction and motherhood, this means that women have limited control over their time. They can hardly decide whether to wait and delay motherhood, because both biology and society are perceived as the sole legitimate owners of women’s time, and they are the only determinants of when “the time has come” to be a mother. Any other relationship between women and time in this context tends to be framed in capitalist terms as a “waste of time,” that is, time that is of no value, leading to devaluation of the present (Lahad, 2016). Thus, the issue of remaining in a state of indecisiveness, with regards to ownership of time, is a significant matter in a society where the pace of life is dictated by capitalist, neoliberal, pronatalist, and heteronormative logics, and in a society that requires people to demonstrate visible “progress” toward the “right” decision at the “right” pace (Amir, 2005). It is also a key issue in a society where women are viewed as essentially in a state of waiting that has been **forced** on them: “Women have always been perceived as expectant figures: they are expecting to be addressed, they are expecting to get their period out of fear that it will not come; they are expecting men to come home from battle or from work; they are expecting their children to grow up; they are expecting to give birth to a new child; or they are expecting menopause” (Rich, 1976, pp. 68).

Studies conducted by Lahad (2016) and Israeli-Nevo (2017) have shed light on the way that waiting or delays while “taking time for myself,” without hurrying to fulfill expectations for the future, can involve an experience of insisting on ownership of time, in a manner that completely disrupts linear perspectives of time as moving toward the “right act” or the “right appearance” of the body. In this sense, the wish of some participants to remain in a state of uncertainty, and to not be classified into categories of “want to be a mother” or “do not want to be a mother,” undermines some of the fundamental logics of contemporary society.

During the group discussions, participants also discussed the various ways by which they can “be a mother.” However, this issue is beyond the scope of the current paper and will be addressed elsewhere. Future studies would benefit from further investigations into the meanings that women who are indecisive about motherhood ascribe to “being a mother.” Findings from such studies may enrich our understanding of the perceptions accompanying indecisiveness about motherhood.

### Summary

In this article, we sought to broaden the limited body of knowledge regarding women who are.

in a state of indecision, self-clarification, and uncertainty about motherhood. We contend that the state of indecision should not be viewed as a passing or idiosyncratic phase, but rather as a state that deserves comprehensive sociological investigation. The state of indecisiveness about motherhood, whether long-term or temporary, can teach us about the perceptions, types of discourses, and cultural understandings regarding fertility, motherhood, decision, and indecision, as well as about the relationship between decisions and inaction.

We demonstrate that while indecisiveness is nurtured by neoliberal rhetoric, it simultaneously undermines that rhetoric, as well as pronatalist, heteronormative, and temporal linear norms. We also show how participation in a group that aims to respect uncertainty can be in itself an island of resistance.

Under neoliberal, capitalist/consumerist, and post-feminist logic in the late modern era, more and more women from different social groups are perceived as equal citizens in the “Republic of Choice” (Ginsburg and Rapp, 1991). Nonetheless, it appears that women’s freedom is still limited with regards to the arena of reproduction and motherhood. That is, women are given the right to choose what society wants them to choose. As long as women’s choices are in line with the hegemonic discourses—and follow the priorities that they dictate and the roles selected for them (e.g., to be “sexually free,” “well-kept,” consumerist, and to live with a spouse as mothers)—women are socially respected, and view themselves as free subjects who are independent, autonomous, and have desires that they are free to realize. However, women encounter a problem when their choices are not in line with the social directives, for example, if they refuse to devote themselves to being “well-kept” or to living with a spouse, particularly with a man. Not only are many women condemned for such choices, but many contemplate these implications alone because it is perceived as “their own choice,” while disciplinary action and denunciation are expected for those who make “wrong choices” (Gill, 2008). Therefore, in the contemporary era, the discourse of “there is no choice” has been replaced with the discourse of “bad choices” (Solinger, 1998).

This pattern is particularly evident in the arena of fertility, natality, and motherhood. Although more women have the option of choosing today than in the past, they are still expected to make “the right choice,” that is, to bear children (Oliver, 2012). In this social context, women’s indecisiveness about motherhood contradicts the neoliberal order of “knowing, deciding, striving, and conquering”; the expectation in post-feminist discourse that the “woman who chooses” will make an appearance; the pronatalist rhetoric that claims there is a “natural transition” to motherhood; and temporal linear discourse in which women are expected to “progress” toward motherhood at the pace of the ticking “biological clock.”

The establishment of a group for women who are deliberating motherhood provides legitimacy for these oppositions. The group sessions created a safe and autonomous space for the participants, in which they could learn that they were not alone in not knowing what they want, and could hear other women who expressed uncertainty about motherhood. In turn, this provided each participant the opportunity to observe the state of indecisiveness from a different perspective, as they struggled to avoid attaching definitive timetables and target dates.

In this sense, the group sessions challenged the individualist, neoliberal order, in which the individual is placed in the center along with their desires, preferences, interests, and goals, which are clearly defined, along with the various means for achieving them. The group sessions also challenged the order that clarification of goals, or determining the most effective means of attaining them, takes place through a rational decision-making process that operates according to a predetermined and fixed timetable. Moreover, the group sessions challenged the cyclical individualist order that praises the individual’s own will, which is meant to spur actions deriving exclusively from personal will. Participation in the group “disrupted” this cyclical order and distinguished between the two components of that order, thus cutting the Gordian knot connecting them.

The presently conducted type of group is not the only forum that can enable women to explore their desires. Other settings can potentially serve the same purpose, such as friendships, intimate relationships, therapeutic spaces, or any other space that gives women an opportunity to be autonomous subjects in themselves, including spaces for isolation and privacy (see *A Room of One’s Own* by Virginia Woolf). Considering that different women have different priorities and paces of life, it is not worthwhile to create a hierarchy of one form while insisting on autonomy (Tietjens-Meyers, 2001).

Nonetheless, this type of group is unique in that it is public and political. Even when such groups are intended for making personal and private decisions, by their very existence, they have the potential to undermine the rigid directives discussed in the current article. Every word said by those women when they talk among themselves about their personal uncertainty that links personal biographies with rigid social structures, plays an active role in breaking down and constructing the “vocabulary that can describe the restraining forces that dictate the lives of most of the world’s population” (Rottenberg, 2018, p. 438). We can only wait and see whether or not these islands of opposition will expand, as additional groups of this nature will emerge and enable more and more women to develop greater autonomy and a sense of ownership over their desires and actions.

A major limitation of this study is that the majority of the women who participated in the group sessions were heterosexual. Inclusion of women who identify as lesbian or bisexual/pansexual may have yielded different findings. Nonetheless, this is an important study that gives voice to women who are deliberating about motherhood, a unique group that has received little attention in both the research literature and the public discourse. The study provides insight into the experience of indecisiveness about motherhood within a familistic and pronatalist context. Our findings expand existing typologies of women’s reproduction decision-making by showing that women can become mothers and still define themselves as undecided about motherhood.

## Data Availability Statement

The original contributions presented in the study are included in the article/supplementary material, further inquiries can be directed to the corresponding author.

## Ethics Statement

The studies involving human participants were reviewed and approved by the departmental ethics committee at Ben-Gurion University of the Negev. The patients/participants provided their written informed consent to participate in this study.

## Conflict of Interest

The authors declare that the research was conducted in the absence of any commercial or financial relationships that could be construed as a potential conflict of interest.

## Publisher’s Note

All claims expressed in this article are solely those of the authors and do not necessarily represent those of their affiliated organizations, or those of the publisher, the editors and the reviewers. Any product that may be evaluated in this article, or claim that may be made by its manufacturer, is not guaranteed or endorsed by the publisher.

## Author Contributions

All authors listed have made a substantial, direct, and intellectual contribution to the work and approved it for publication.

## Footnotes

^1^However, note that Fogiel-Bijaoui (2020) points out that this process varies according to social group; hence she called it ‘differentiated individualism.’

^2^While heterosexuality was not criteria for selection, the vast majority of the women who approached this group were heterosexual. Additional groups that were conducted between the years 2016–2021included also women who identified themselves as lesbians or bisexuals. These groups were not included in the current analysis and will be addressed elsewhere.

^3^The words in bold signify that the woman herself raised her voice or stressed certain words.

^4^This is in contrast to women coping with fertility problem and for that reason are treated with relative compassion – both because they show a desire to be mothers and make sacrifices if they choose to proceed with fertility treatments, and because they are involuntarily childless.
